# Small bowel segment with Meckel’s diverticulum volvulus related to short mesodiverticular band: a case report

**DOI:** 10.1186/s13256-023-03844-x

**Published:** 2023-03-25

**Authors:** Michał Zieliński, Patryk Kaczor, Grzegorz Jarczyk, Marek Jackowski

**Affiliations:** Department of General, Gastroenterological and Oncological Surgery, Collegium Medicum Nicolaus Copernicus University, 53-59 Św. Józefa St, 87-100 Toruń, Poland

**Keywords:** Meckel’s diverticulum, Mesodiverticular band, Small bowel obstruction, Virgin abdomen, Internal hernia, Case report, Laparotomy, Small intestine volvulus

## Abstract

**Background:**

Meckel’s diverticulum is a remnant of the omphalomesenteric duct and occurs in only about 2% of people. Mesodiverticular band is the congenital remnant of the vitelline artery and is an even less often occurring phenomenon.

**Presented case:**

We present the case of a 56-year-old Caucasian male who was admitted to the emergency department with a very intense, sudden abdominal pain, without past abdominal surgery history. Contrast enhanced computed tomography showed a possibly ischemic closed loop of the small intestine. Urgent laparotomy was performed, during which bloody content in the peritoneal cavity and torsed loop of the small intestine with Meckel’s diverticulum were found. The bowel loop and Meckel’s diverticulum were ischemic. At the tip of Meckel’s diverticulum there was a broken fibrous band extending to mesentery with pulsating artery. We did segmental resection of small intestine including Meckel’s diverticulum and primary end-to-end anastomosis. The patient had an unremarkable postoperative hospital stay and was discharged home after 5 days.

**Conclusion:**

In our case, we describe a patient with the volvulus of a segment of small bowel and Meckel’s diverticulum, which eventually led to small bowel obstruction and ischemia. It was a very rare case that required urgent surgical treatment.

## Background

There are many potential etiologies of small bowel obstruction (SBO). The most common cause of SBO are post-surgical adhesions [1]. However, it may also occur in patients without any previous surgical interventions or other known pathology, in patients with a so-called virgin abdomen (VA). Reported etiologies of SBO-VA include congenital adhesions, malignant or benign tumors, and a much less common cause: Meckel’s diverticulum (MD) [2].

MD is a remnant of the omphalomesenteric duct and is the most common congenital anomaly of the gastrointestinal tract (GI). MD occurs in about 2% of people, and is usually 3–5 cm long with a 4–6% lifetime risk of becoming symptomatic. The most common complications are bleeding, diverculitis, obstruction, and perforation [3]. Even less often it is accompanied by mesodiverticular band (MBD), which is the congenital remnant of the vitelline artery. MDB increases the frequency of complications, especially in the form of incarceration of internal hernia or small intestine volvulus [4].

We present a case of SBO caused by torsion of MD and segment of small bowel, resulting in gangrenous change related to short MDB. Additionally, a broken MDB with arterial vessel caused bleeding into the peritoneal cavity.

## Case report

**Fig. 1:**
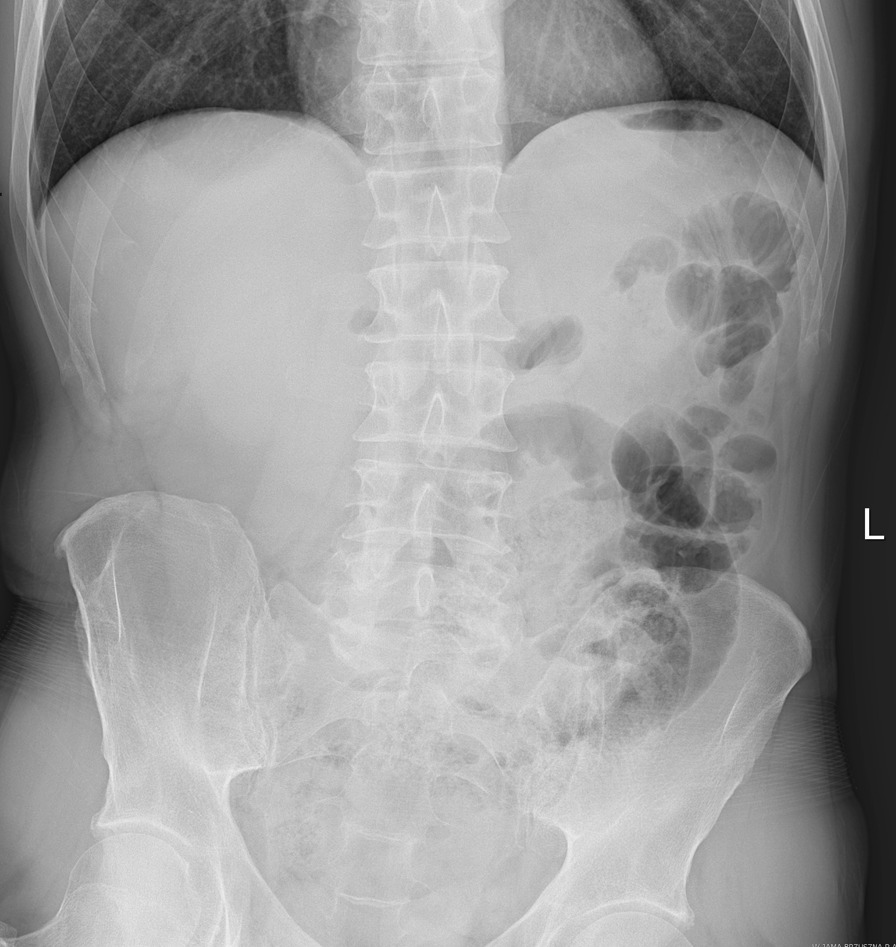
Abdominal X-ray, dilated loop of the small intestine in the lower abdomen

**Fig. 2:**
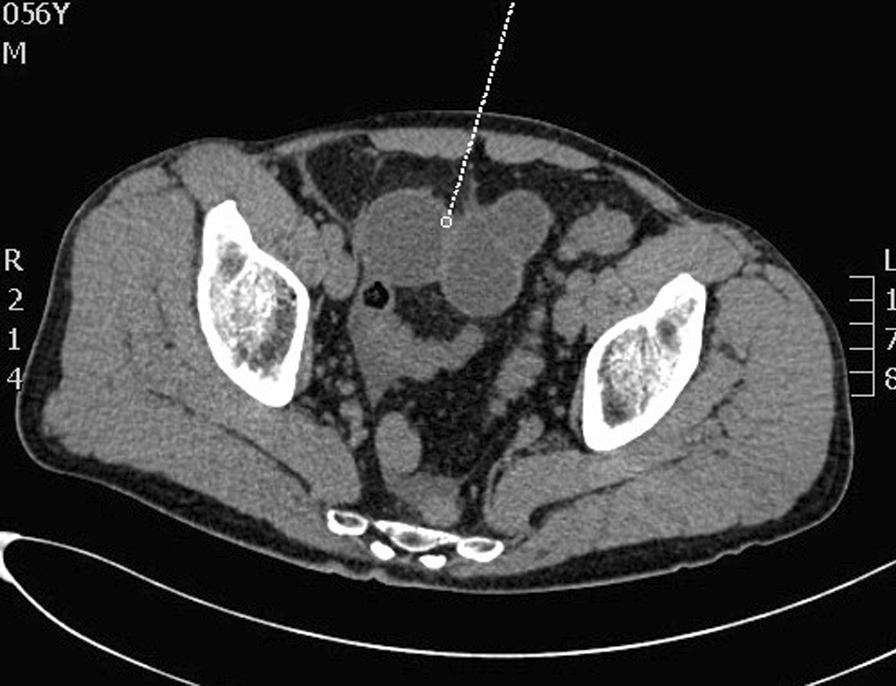
CT scan

**Fig. 3:**
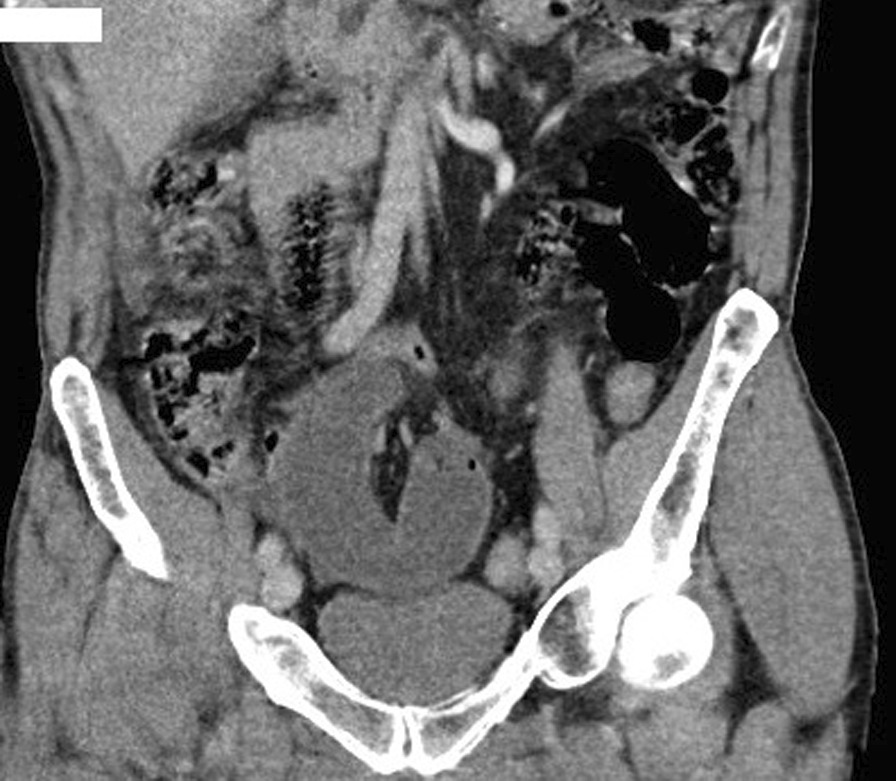
CT scan. White line refers Meckel's diverticulum

**Fig. 4:**
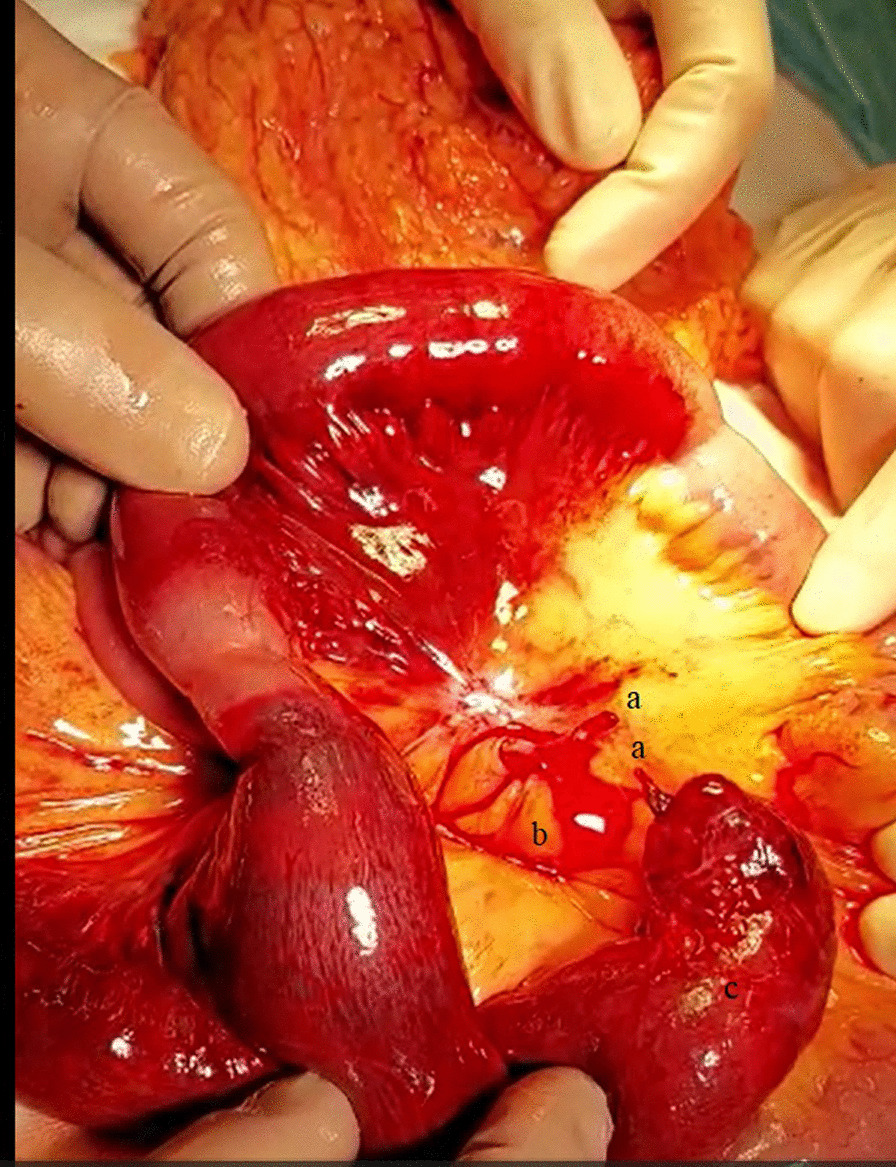
Intraoperative view, **a**—ends of MDB; **b**—mesentery; **c**—MD

**Fig. 5:**
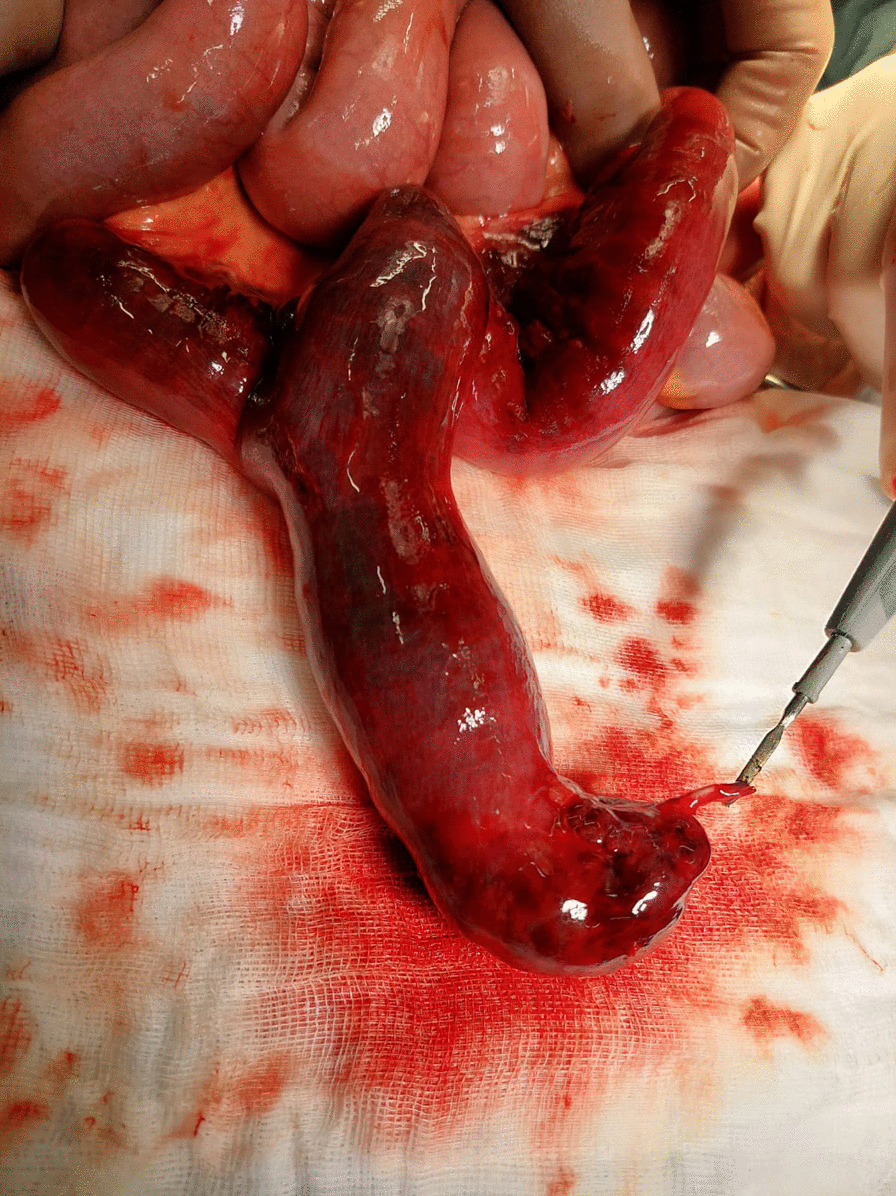
MD with part of MDB

**Fig. 6:**
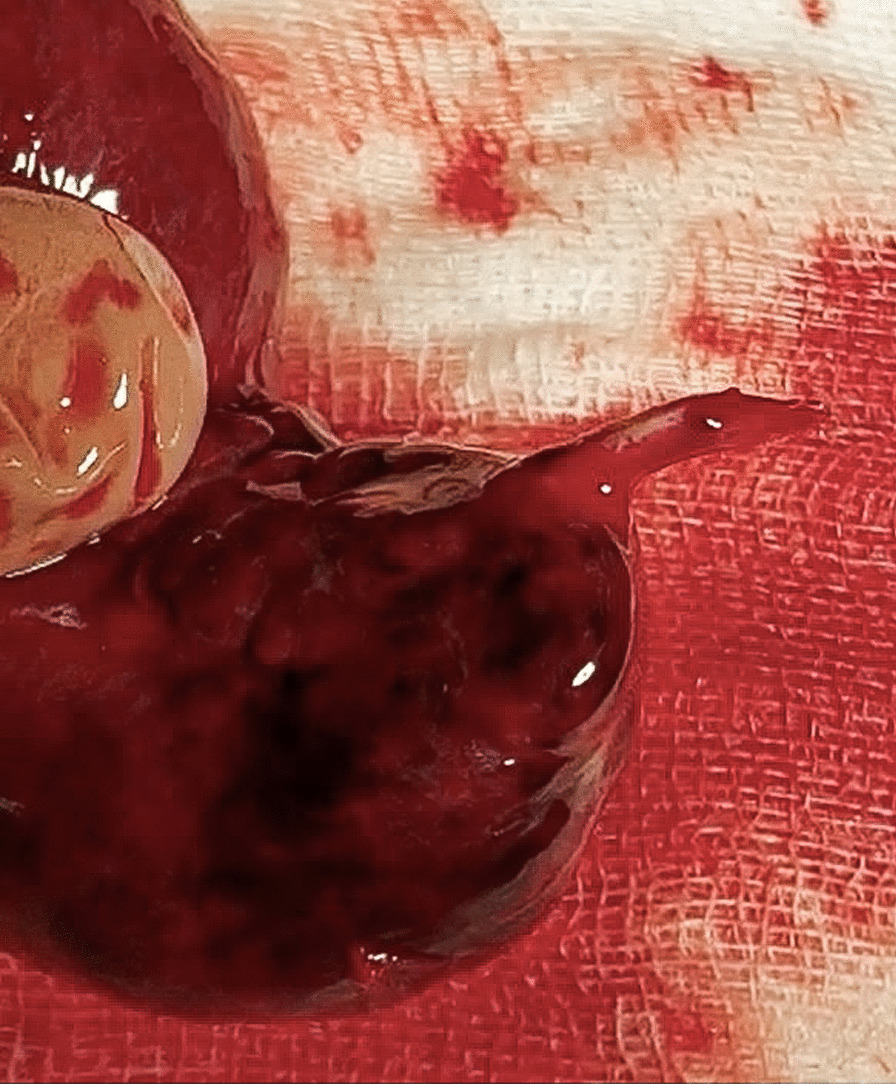
MD with part of MDB

A 56-year-old Caucasian male was admitted to the emergency department (ED) with severe abdominal pain for 4 hours, which started suddenly. His significant past medical history included only the surgery of his left shoulder. He reported no abdominal surgeries, chronic diseases or permanent medications. We asked him a few more questions and he noted abdominal pain occurring every few months for 2–3 days since he was 20. The physical examination showed normal vital signs, the abdomen was flat with severe palpatory pain, peritoneal symptoms were present, and there were no bowel sounds. Laboratory tests showed isolated leukocytosis (16.62 × 10^3^/µL) with no other abnormalities. In the plain abdominal X-ray only a dilated loop of the small intestine in the lower abdomen was visible (Fig. 1), without any sign of gastrointestinal obstruction. Due to the severe abdominal pain, contrast enhanced computed tomography (CT) was performed. CT scan (Figs. 2, 3) showed a widened to 40 mm loop of the small intestine in the lower abdomen, which was filled with liquid content. On the left side, at the height of the promontory, there was a sudden narrowing of the intestinal lumen and a transition to another fragment of the intestine located in the left iliac fossa, forming a closed loop 123 mm long. The wall from the side of the mesentery and the adipose tissue was obliterated, and there was a trace of fluid adjacent to the loop, with possible ischemia of the loop. Patient was qualified for surgical treatment. During exploratory laparotomy, we found a small amount of bloody content in the peritoneal cavity and dilated small intestine proximal to the twisted loop of the small intestine including MD about 15 cm long. Twisted loop and MD were cyanotic and hyperemic. MD was located on the antimesentric border of the ileum, about 80 cm proximal to the ileocecal valve. There was a broken fibrous band at the tip of the MD extending to the mesentery, which contained pulsating artery (Figs. 4, 5, 6). The probable mechanism of intestinal volvulus was an internal herniation due to MDB. We performed segmental intestine resection (with MD) and primary end-to-end anastomosis. After the surgery, resected MD was cut open, revealing bloody content and a “double bottom” containing thrombus.

There were no postoperative complications. The patient was discharged home 5 days after surgery. The histopathological examination revealed Meckel’s diverticulum with hemorrhagic necrosis, with no mention of ectopic tissue.

## Discussion

MD is the remnant of omphalomesenteric duct and it is the most common congenital malformation of GI tract. MD is present in approximately 2% of people. The majority of MD are asymptomatic. Complications are described only in 4–6% of cases, which most commonly include hemorrhage, intestinal obstruction, diverticulitis, and perforation [3]. It is worth remembering that MD may contain ectopic tissue and the risk of carcinogenesis is 70% higher than in “normal” small intestine [5].

MD rarely comes together with MDB, which is a remnant of a vitelline artery. It carries arterial blood supply to MD. The MDB might be patent or non-patent arterial band [6].

The presence of MDB increases risk of serious complications associated with MD and it is related to higher mortality.

This embriologic band extending from mesentery to the tip of MD creates a gate for internal hernia. It is a rare situation to have an axial torsion of MD and concurrent segmental small bowel volvulus [4, 6, 7].

As mentioned above, MD can cause GI bleeding due to ectopic tissue. In our case, despite the lack of histologically different ectopic tissue, bleeding into the diverticulum also occurred as a result of the presence of MDB and the patent artery. What is more, there are cases of MDB rupture causing peritoneal bleeding [8]. The exact time of rupture of MBD in our case is unknown. Fortunately, this was not associated with clinically significant blood loss.

Bamarni *et al*. claimed in their 2021 study that complications related to MDB may occur at any age, in contrast to usual presentation of MD in the pediatric population [4]. Our patient was 56 years old, which is well above the average age of onset of MD complications.

MDB, as well as peritoneal adhesions, are usually not radiologically detectable. However, giant MD might create a visible blind loop [7]. It is definitely more difficult to detect a small diverticulum, which can also cause serious complications. CT scan in our case showed a blind loop, which corresponded to MD. In our case, from the available preoperative tests, only CT had a positive diagnostic value. Neither laboratory tests nor abdominal X-ray were specific for this acute surgery condition. Furthermore, our patient had been experiencing recurrent non-specific abdominal pain for many years. Eventually patient was admitted to the ED with severe abdominal pain and “virgin abdomen.” Physicians should be alert not to forget about MD in such cases.

There are still no clear guidelines for the management of uncomplicated MD [9, 10], but it seems that the discovery of large MD and the presence of MDB should be an indication for removal to prevent any further complications and possible life-threatening conditions. Moreover, there are no exact data on the prevalence of MDB. So far only a few similar cases of intestinal volvulus have been described in the literature [4, 7, 11–13].

## Conclusion

SBO is a common reason for admission to the surgical department. In the differential diagnosis of the causes of SBO, we should remember rare causes such as MDB. Without CT imaging, the patient would have been treated conservatively, which in the case of complicated MD could have led to fatal complications, including death.

Early diagnosis and immediate surgical treatment are crucial in this case. We should keep in mind that this condition is very rare, which is the main reason for this publication.

## Data Availability

Data available on request (corresponding author email: zielinski.michals@gmail.com).

## Abbreviations

MD: Meckel’s diverticulum
MDB: Mesodiverticular band
SBO: Small bowel obstruction
VA: Virgin abdomen
ED: Emergency department
GI: Gastrointestinal tract
